# Can digital health literacy promote residents’ physical and mental health? An empirical analysis based on Chinese General Social Survey

**DOI:** 10.3389/fpubh.2025.1654330

**Published:** 2025-10-17

**Authors:** Pingqiang Wei, Haigang Jia

**Affiliations:** ^1^School of Literature and Journalism, Xihua University, Chengdu, China; ^2^College of Marxism, Nanjing Forestry University, Nanjing, China

**Keywords:** digital health literacy, physical health, mental health, participate in physical exercise, social interaction

## Abstract

With the rapid development of digital technology, the role of digital health literacy in improving the physical and mental health of residents has become increasingly prominent. Based on the survey data of CGSS2021, this study selected 1805 valid samples. Multiple linear regression model and Bootstrap mediating effect test were used to explore the impact of physical exercise, social interaction and digital health literacy on residents’ physical and mental health. The results show that: (1) Digital health literacy has a significant positive impact on residents’ physical and mental health (physical health: *β* = 0.146, *p* < 0.05; mental health: *β* = 0.752, *p* < 0.01); (2) Participation in physical exercise and social networks have a mediating role in the impact of digital health literacy on residents’ physical and mental health, and have a chain mediating role; (3) Digital health literacy promotes residents’ physical and mental health, but there is heterogeneity. In rural areas and women with low or normal BMI, the improvement of digital health literacy has a significant effect on physical health. In the male group of urban residents, the improvement of digital health literacy has a significant effect on mental health. Based on this, we propose: First, strengthen the promotion of digital health literacy education, especially the digital health literacy level of residents and women in rural areas. Second, encourage residents to actively participate in physical exercise, physical exercise as a link to promote the physical and mental health of residents. The third is to promote the development of social interaction activities, and various organizations expand social support networks to provide strong support for residents’ mental health.

## 1 Introduction

Digital health literacy is the extension and upgrading of health literacy in the digital age. It originated from health literacy and was initially defined as the ability of individuals to acquire, understand and use health information to maintain and improve health (1). With the development of information technology, NORMAN and SKINNER proposed the concept of “e-health literacy” in 2006, emphasizing the ability to obtain and apply health information from electronic resources (2). On this basis, the academic community further proposes a more comprehensive concept of “digital health literacy” to adapt to the reality of the continuous evolution of digital technology. Although the connotation of digital health literacy has not yet reached a consensus, the relevant research mainly presents two viewpoints: one regards it as the integration of digital literacy and health literacy, emphasizing the ability of individuals to obtain health information and solve health problems through digital devices (3); the other is that it is the expansion of health literacy in the digital environment, including not only the ability to find, evaluate and apply health information, but also the ability of individuals to interact with digital content, share information and actively participate in health communication (4). In addition, the research topic has gradually shifted from the early application of mobile health technology to diversification, focusing on the health applications of telemedicine, artificial intelligence and wearable devices at the technical level (5). Focus on the special needs and obstacles of the older adult, patients with chronic diseases, pregnant women and other groups at the population level (6, 7); at the social level, the digital divide has become a core issue, highlighting the inequality of technology acquisition and application caused by socioeconomic status and age differences (8, 9).

With the continuous development of Internet technology, digitization, digital intelligence and AI have become the inevitable trend of social development. Through digital technology, we can broaden the dissemination of health knowledge, empower the reform of health care industry, and promote the continuous development of digital health (10). As of December 2024, the number of Internet medical users in China reached 418 million, and the supply of digital services such as Internet medical care continued to increase. With the innovative application of online medical insurance purchase and medical big model, the level of Internet medical services has been significantly improved. At present, there are 3,340 Internet hospitals in China, providing more than 100 million Internet diagnosis and treatment services each year. Online drug purchase, medical insurance payment, and instant delivery not only further reduce the burden of drug purchase by the masses, but also significantly improve the convenience of drug purchase (11). This shows that Internet medical treatment has been widely used and accepted in China, and more and more residents begin to use Internet medical treatment to meet their health needs. This huge user group provides a solid foundation for the development of the Internet medical industry, and also reflects the deepening penetration of digital services in the health field, becoming an indispensable part of residents’ health management. However, when residents accept the good life brought by digital health information, because of the diversity of information sources and the uneven quality of information, it is difficult for residents to distinguish the authenticity and easy to be misled, resulting in anxiety and concern. On the other hand, residents who are not familiar with digital technology or have low digital literacy experience technical barriers when using services such as Internet medical care, such as complex processes, unfamiliar interfaces, privacy concerns, etc., resulting in frustration and rejection (12, 13).

As a key ability for individuals to acquire, understand, evaluate and apply digital health information, digital health literacy has become a core element to bridge the health gap and empower residents to manage their own health (14–16). Research shows that digital health literacy not only directly promotes residents’ physical and mental health by improving the efficiency of health information acquisition and the scientific nature of decision-making (17–19), but also is more likely to have an indirect effect by participating in physical exercise and social interaction (20–22). However, there is still uncertainty in its mechanism of action. In particular, whether physical exercise and social network play an intermediary role in digital health literacy and residents’ physical and mental health remains to be further explored.

Chinese General Social Survey (CGSS) began in 2003. It systematically and comprehensively collects data at multiple levels of society, community, family and individual, summarizes the trend of social change, discusses issues of great scientific and practical significance, promotes the opening and sharing of scientific research in China, provides data for international comparative research, and serves as a multidisciplinary economic and social data collection platform. At present, CGSS data has become the most important data source for studying Chinese society and is widely used in scientific research, teaching and government decision-making. Therefore, based on the CGSS data survey, this study empirically analyzes and answers the following questions: What is the relationship between digital health literacy and residents’ physical and mental health? Is this relationship different due to individual characteristics? Does participation in physical exercise and social network play an intermediary role between digital health literacy and residents’ physical and mental health? On this basis, the mechanism of the impact of digital health literacy on the physical and mental health of residents is further analyzed.

## 2 Literature review and research hypothesis

Physical and mental health is the core foundation of residents’ quality of life and the key premise of human capital accumulation (23). As an individual’s ability to obtain and apply health information through digital technology (24), digital health literacy is not only a tool to obtain health information (25), but also an important way to alleviate health inequality (26). It can help the older adult to optimize health behaviors and reduce health anxiety (27). Its absence may expose residents to health risks and hinder the exercise of medical self-determination and legal capacity due to the inability to effectively access or utilize digital health resources (28). At the same time, 17 factors such as age, education level and social support were significantly correlated with residents’ digital health literacy (29). Among them, the digital health literacy of the older adult was more affected by socio-demographic factors, electronic equipment factors, and use and social support factors (30); at the level of health information acquisition behavior, the level of personal digital health literacy is directly related to the frequency of searching for health information (31), and emotional intelligence can further regulate the relationship between digital literacy and online health use (32). These factors form a differentiated role path in physical health management and mental health maintenance by changing the ability of individuals to acquire, understand and use digital health resources.

Research on the validation of the impact of digital health literacy. At the physical health level, a survey based on 19,231 people showed that the higher the digital health literacy, the higher the level of individual physical activity (33), and the use of YouTube to obtain health content can further strengthen the exercise intention (34). At the level of mental health, digital mental health intervention has been shown to significantly reduce depression and anxiety symptoms (35), and digital health literacy itself can alleviate technical anxiety (13) and health anxiety (36). This protective effect is particularly prominent in the older adult group (37).

Research on the impact mechanism of digital health literacy on health. By strengthening the ability of health information processing, digital health literacy enables individuals to efficiently screen, understand medical knowledge and transform it into scientific health behaviors, which can directly promote the phased transformation of physical exercise, and play an indirect effect with the help of behavioral process and self-efficacy (38); the improvement also significantly broadens the access to health resources from online consultation to intelligent monitoring. The comprehensive development of cognition, operation and evaluation dimensions significantly drives the intention of health exercise behavior (39), and is positively correlated with the viewing experience of health content (40). In addition, digital health literacy optimizes the effect of chronic disease intervention by enhancing self-health management awareness, and extends the value of social support networks and psychological intervention channels constructed by digital platforms in the field of mental health to effectively alleviate anxiety and depression (41).

In summary, this study defines digital health literacy as the comprehensive ability of individuals to dynamically acquire, understand, evaluate, apply health information and participate in health interaction through digital technology, covering three core dimensions of technical operation, cognitive criticism and application decision-making. In order to meet the requirements of this conceptual scheme, this study will select Chinese General Social Survey data for empirical research. In the dimension of health impact, digital health literacy affects physical and mental health through the triple mechanism of information resource acquisition path, information processing path and social psychological path. Based on the above mechanism, this study proposes the following research hypotheses:

*H1a:* Digital health literacy has a positive impact on residents' physical health.

*H1b:* Digital health literacy has a positive impact on residents' mental health.

*H2a:* Participation in physical exercise plays an intermediary role in digital health literacy and residents' physical health.

*H2b:* Participation in physical exercise plays an intermediary role in digital health literacy and residents' mental health.

*H3a:* Social network plays an intermediary role in digital health literacy and residents' physical health.

*H3b:* Social network plays a mediating role in the relationship between digital health literacy and residents' mental health.

*H4a:* Participation in physical exercise, subjective well-being, and social network have a chain mediating effect in digital health literacy on residents' physical health.

*H4b:* Participation in physical exercise, subjective well-being, and social network have a chain intermediary in digital health literacy to residents' mental health.

## 3 Materials and methods

### 3.1 Data sources

Chinese General Social Survey is the first national, comprehensive and continuous large-scale social survey project in China. The survey was conducted jointly by Renmin University of China and academic institutions across the country. Since 2003, a sample survey of more than 10,000 families across the country has been conducted every year. This study used 2021 data released free of charge by CGSS.^1^ CGSS2021 completed a total of 8,148 valid samples nationwide, including 700 variables. Considering that the key variables of this study mainly involve digital health literacy, residents’ physical and mental health, participation in physical exercise, and social networks, after screening and preprocessing the missing values and invalid answers of the above variables, 1805 valid observations were finally retained.

### 3.2 Variable measurement

According to the CGSS2021 survey data, this study selected physical health status and mental health status as dependent variables, selected digital health literacy as independent variables, selected participation in physical exercise and social network as intermediary variables, and selected gender, age, place of residence, political identity, religious belief, BIM index, social equity cognition, job satisfaction, and education level as control variables. The data of each variable are provided by the answers to the CGSS2021 survey questions, and all variables are shown in Table 1.

**Table 1:** Variable assignment.

Variable	Survey questions used as measurement indicators	The assignment of variables
Digital health literacy	Do you often search for information about healthy lifestyles online?	Never = 1, rarely = 2, sometimes = 3, often = 4, very often = 5
	Do you often search for anxiety, stress-related or similar information online?	
	Do you often search the Internet for information about vaccination?	
	Does the information on the Internet have a positive impact on my health behavior?	Very disagree = 1, disagree = 2, agree or disagree can be = 3, agree = 4, very agree = 5
	Does the information on the Internet help me understand the doctor’s advice and treatment information?	
	Does the Internet help to determine whether you need to go to the hospital?	
	Does the Internet help to confirm whether the doctor has given appropriate advice?	
	It is not easy to identify whether the health information on the Internet is reliable.	
Physical health	What do you think your current health status is?	Very unhealthy = 1, relatively unhealthy = 2, generally = 3, relatively healthy = 4, very healthy = 5
Mental health	How often have you felt depressed or depressed over the past four weeks?	Always = 1, often = 2, sometimes = 3, rarely = 4, never = 5
Participating in the physical exercise	In the past 1 year, have you often participated in physical exercise in your free time?	Never = 1, times a year or less = 2, times a month = 3, times a week = 4, days = 5
Social networking	How often do you have social entertainment activities with your neighbors (such as visiting each other, watching TV together, eating, playing cards, etc.)?	Never = 1, once a year or less = 2, several times a year = 3, approximately once a month = 4, several times a month = 5, 1 to 2 times a week = 6, almost every day = 7
	How often do you have social and recreational activities with other friends (such as visiting each other, watching TV together, eating, playing cards, etc.)?	
Gender	Female = 0, male = 1	
Age	What is the year of your birth date?	Actual numerical value
Place of abode	What is your current household registration status?	Rural = 0, urban = 1
Political identity	What is your current political outlook?	Others = 0, Chinese Communists = 1
Religious belief	What is your religious belief?	Non-religion = 0, religion = 1
BMI index	Indicators calculated based on the weight and height data of the individuals surveyed.	
Social justice recognition	In general, do you think today’s society is unfair?	Complete unfairness = 1, comparative unfairness = 2, unfairness but not unfairness = 3, comparative fairness = 4, complete fairness = 5
Job satisfaction	In general, are you satisfied with your current job?	Very dissatisfied = 1, not very satisfied = 2, general = 3, relatively satisfied = 4, very satisfied = 5
Education level	What is your current highest education level?	No education = 1, private school, literacy class = 2, primary school = 3, junior high school = 4, vocational high school = 5, ordinary high school = 6, secondary school = 7, technical school = 8, junior college (adult higher education) = 9, junior college (formal higher education) = 10, undergraduate (adult higher education) = 11, undergraduate (formal higher education) = 12, postgraduate and above = 13

#### 3.2.1 Dependent variable

The dependent variables of this study are physical health status and mental health status. About physical health, drawing on the existing research results, select the CGSS2021 questionnaire survey question “Do you think your current physical health status is?” (56). The answer options are “very unhealthy,” “relatively unhealthy,” “general,” “relatively healthy,” “very healthy,” and are assigned 1, 2, 3, 4, and 5, respectively. The higher the score, the better the physical health. On the mental health status, drawing on existing research results, select the CGSS2021 questionnaire survey questions “In the past 4 weeks, how often do you feel depressed or depressed?” (42). The answer options are “always,” “often,” “sometimes,” “rarely,” “never,” and are assigned 1, 2, 3, 4, and 5, respectively. The higher the score, the better the mental health status.

#### 3.2.2 Independent variables

The independent variable of this study is digital health literacy. On digital health literacy, drawing on existing research results, combined with the corresponding indicators of CGSS, this study constructs residents’ digital health literacy indicators from three dimensions and eight problems: digital health information acquisition, cognitive critical health information, and skills of applying information to solve health problems (20). Because 8 questions have different categories of answers, in order to better judge and measure the ranking of digital health literacy, this study uses the entropy weight TOPSIS method to determine the weight of digital health literacy indicators, and calculates the digital health literacy score for comprehensive evaluation (see the Appendix for specific method steps) (43, 44). The value range of digital health literacy score is 0–1. The larger the value is, the closer the digital health literacy is to the optimal solution, that is, the larger the value is, the higher the level of digital health literacy is.

#### 3.2.3 Mediating variables

The mediating variables of this study are physical exercise and social network. On participating in physical exercise, drawing on existing research results, select the CGSS2021 questionnaire survey question “In the past 1 year, do you often participate in physical exercise in your free time?” (45). The answer options are “daily,” “several times a week,” “several times a month,” “several times a year or less,” “never,” and the answer is inversely assigned. The score is 1–5. The higher the score, the higher the frequency of physical exercise. Regarding the social network, based on the existing research results, the CGSS2021 questionnaire survey questions “the frequency of social entertainment activities with neighbors” and “the frequency of social entertainment activities with other friends” were selected (46, 47), and the answers were reversely reverse-coded on a 7-point scale. In this study, the average value of the answers to the above two questions is used to measure the social network. The higher the score, the higher the frequency of social interaction.

#### 3.2.4 Control variables

In order to eliminate the influence of confounding factors on residents’ physical and mental health, this study selected gender, age, place of residence, political identity, religious belief, BIM index, social justice cognition, job satisfaction and education level as control variables.

### 3.3 Method

#### 3.3.1 Multiple linear regression model

This study used a multiple linear regression model to explore the impact of digital health literacy on residents’ physical and mental health. The mathematical equation of the multivariate linear regression model is shown in Equation 1:


(1) $$Y=\beta_{0}+\beta_{1}X_{1}+\beta_{2}X_{2}+\beta_{3}X_{3}+\beta_{4}X_{4}+\in$$

In the Equation 1, *Y* is the dependent variable, representing the physical and mental health of residents. X1 is the independent variable, representing digital health literacy. X2 is the control variable. X3 is an intermediary variable, representing participation in physical exercise. X4 is an intermediary variable, representing the social network. $\beta_{0}$ is the intercept term, which indicates the baseline value of residents’ physical and mental health predicted by the model when all independent variables are zero. $\beta_{1}$ - $\beta_{4}$ are regression coefficients, indicating the strength of the influence of each variable on the dependent variable *Y*. Among them, $\beta_{1}$ is the focus of this study, indicating the direct impact of digital health literacy on residents’ physical and mental health. ϵ is the error term, which represents the impact of other factors that have not been observed in the model on the physical and mental health of residents.

#### 3.3.2 Bootstrap method to estimate the mediating effect model

This study uses the Bootstrap method to estimate the mediating effect, to test the significant effect of the mediating effect, and finally to verify whether participating in physical exercise and social network significantly mediate the impact of digital health literacy on residents’ physical and mental health. The total effect equation of the independent variable to the dependent variable is shown in Equation 2:


(2) Y=cX+ϵ

In the Equation 2, Y is the dependent variable, X is the independent variable, c is the total effect coefficient, and ϵ is the random error term. Considering the mediating variable, the equation of the independent variable to the mediating variable is shown in Equation 3:


(3) $$M=\mathrm{aX}+\epsilon_{1}$$

In the Equation 3, M is the intermediary variable, a is the effect coefficient of the independent variable to the intermediary variable, and $\epsilon_{1}$ is the random error term. The equation of the mediating variable and the independent variable to the dependent variable is shown in Equation 4:


(4) $$Y=c\prime X+\mathrm{bM}+\epsilon_{2}$$

In the Equation 4, c′ is the direct effect coefficient of the independent variable to the dependent variable, b is the effect coefficient of the intermediary variable to the dependent variable, and $\epsilon_{2}$ is the random error term. The mediating effect is the indirect effect, which can be expressed as a × b. The total effect c can be decomposed into direct effect c′ and indirect effect a × b, that is, c= c′ +a × b.

The mediating effect was tested using the Bootstrap sampling method. The Bootstrap sampling test refers to whether the 95% confidence interval of the regression coefficient a*b includes the number 0; if the 95% confidence interval does not include the number 0, it shows that it has a mediating effect; if the 95% confidence interval includes the number 0, it means that there is no mediating effect.

#### 3.3.3 Grouped regression model

This study analyzes whether there are differences in the impact of digital health literacy on residents’ physical and mental health from gender, place of residence and BMI index by grouping regression model. Assuming that there are two groups of samples, the first group of sample equations are shown in Equation 5:


(5) $$Y_{1i}=\alpha_{1}+\beta_{1}X_{1i}+\epsilon_{1i}$$

In the Equation 5, $Y_{1i}$ is the dependent variable of the i th individual in the first group of samples, $\alpha_{1}$ is the intercept term of the first group, $\beta_{1}$ is the regression coefficient of the independent variable $X_{1i}$ of the first group to the dependent variable, and $\epsilon_{1i}$ is the error term, indicating other random factors affecting $Y_{1i}$ except $X_{1i}$ . For the second group of sample equations, as shown in Equation 6:


(6) $$Y_{2i}=\alpha_{2}+\beta_{2}X_{2i}+\epsilon_{2i}$$

In the Equation 6, $Y_{2i}$ is the dependent variable of the i th individual in the second group of samples, $\alpha_{2}$ is the intercept term of the second group, $\beta_{2}$ is the regression coefficient of the second group independent variable $X_{2i}$ to the dependent variable, and $\epsilon_{2i}$ is the error term, which indicates the random factors affecting $Y_{2i}$ other than $X_{2i}$ .

## 4 Results

### 4.1 Descriptive statistical results

The descriptive results of each variable are shown in Table 2. It can be seen from Table 2 that the effective sample data is 1805, the score range of the dependent variable digital health literacy is 0–1, and the mean value is 0.383 (*SD* = 0.142), indicating that most of the sample digital health literacy is not high. The score range of the independent variable physical health is 1–5, and the mean value is 3.658 (*SD* = 0.990). The score range of the independent variable mental health is 1–5, and the mean value is 4.027 (*SD* = 1.026), indicating that most of the samples have good physical and mental health.

**Table 2:** Descriptive results of each variable.

Variable	*M*	*SD*	*Min*	*Max*	*N*
Digital health literacy	0.383	0.142	0	1	1805
Physical health	3.658	0.990	1	5	1805
Mental health	4.027	1.026	1	5	1805
Participating in the physical exercise	3.012	1.535	1	5	1805
Social networking	4.886	0.739	1	7	1805
Gender	0.452	0.498	0	1	1805
Age	45.479	16.568	18	88	1805
Place of abode	0.448	0.497	0	1	1805
Political identity	0.117	0.322	0	1	1805
Religious belief	0.031	0.173	0	1	1805
BMI index	22.820	3.714	8.294	40.428	1805
Social justice recognition	3.429	0.920	1	5	1805
Job satisfaction	3.200	0.559	1	5	1805
Education level	6.284	3.437	1	13	1805

### 4.2 Multiple linear regression model results

In order to explore the impact of digital health literacy on residents’ physical health and mental health, four models were constructed in this study. Among them, model 1 incorporates control variables; on the basis of model 1, model 2 adds participating in physical exercise factors; on the basis of model 2, model 3 adds social network factors; on the based of model 3, model 4 adds digital health literacy factors and makes a collinearity diagnosis. The multiple linear regression results of the influencing factors of residents’ physical and mental health are shown in Table 3.

**Table 3:** Multiple linear regression results of influencing factors of residents’ physical and mental health.

Variable	Model 1		Model 2		Model 3		Model 4					
	Physical health	Mental health	Physical health	Mental health	Physical health	Mental health	Physical health	Collinearity diagnostics		Mental health	Collinearity diagnostics	
								VIF	Tolerability		VIF	Tolerability
Gender	0.063 (0.044)	0.126** (0.048)	0.065 (0.044)	0.127** (0.048)	0.068 (0.044)	0.130** (0.048)	0.071 (0.044)	1.053	0.950	0.115* (0.048)	1.053	0.950
Age	−0.017** (0.002)	−0.001 (0.002)	−0.017** (0.002)	−0.001 (0.002)	−0.018** (0.002)	−0.001 (0.002)	−0.017** (0.002)	1.525	0.656	−0.003 (0.002)	1.525	0.656
Place of abode	−0.040 (0.048)	0.057 (0.052)	−0.064 (0.048)	0.042 (0.052)	−0.059 (0.048)	0.046 (0.052)	−0.060 (0.048)	1.241	0.806	0.054 (0.052)	1.241	0.806
Political identity	0.075 (0.073)	0.210** (0.079)	0.071 (0.073)	0.208** (0.079)	0.068 (0.072)	0.204** (0.079)	0.065 (0.072)	1.181	0.847	0.218** (0.079)	1.181	0.847
Religious belief	−0.134 (0.125)	−0.191 (0.136)	−0.140 (0.124)	−0.194 (0.136)	−0.122 (0.124)	−0.180 (0.136)	−0.127 (0.125)	1.011	0.989	−0.151 (0.135)	1.011	0.989
BMI index	−0.005 (0.006)	0.009 (0.007)	−0.005 (0.006)	0.009 (0.007)	−0.005 (0.006)	0.009 (0.007)	−0.005 (0.006)	1.064	0.940	0.008 (0.006)	1.064	0.940
Social justice recognition	0.096** (0.024)	0.169** (0.026)	0.092** (0.024)	0.166** (0.026)	0.087** (0.024)	0.162** (0.026)	0.087** (0.024)	1.026	0.974	0.160** (0.026)	1.026	0.974
Job satisfaction	0.150** (0.039)	0.205** (0.043)	0.147** (0.039)	0.203** (0.043)	0.146** (0.039)	0.202** (0.043)	0.146** (0.039)	1.027	0.974	0.201** (0.042)	1.027	0.974
Education level	0.028** (0.008)	0.009 (0.009)	0.023** (0.008)	0.006 (0.009)	0.021* (0.008)	0.004 (0.009)	0.020* (0.008)	1.768	0.566	0.007 (0.009)	1.768	0.566
Participating in the physical exercise			0.052** (0.015)	0.032 (0.016)	0.047** (0.015)	0.027 (0.016)	0.045** (0.015)	1.150	0.869	0.038* (0.016)	1.150	0.869
Social networking					0.081** (0.030)	0.067* (0.032)	0.080** (0.030)	1.049	0.953	0.073* (0.032)	1.049	0.953
Digital health literacy							0.146* (0.164)	1.173	0.853	0.752** (0.178)	1.173	0.853
*N*	1805	1805	1805	1805	1805	1805	1805			1805		
Constant	3.552** (0.221)	2.485** (0.241)	3.466** (0.222)	2.433** (0.242)	3.920** (0.277)	2.804** (0.302)	3.847** (0.288)			3.177** (0.313)		
Adjusted R^2^	0.142	0.055	0.147	0.056	0.150	0.058	0.150			0.067		

*p < 0.05, **p < 0.01, standard error in parentheses.

Model 1 is the basic control variable model. The results show that gender has a significant positive effect on mental health. The mental health level of women is better than that of men, but it has no significant effect on physical health. Ageing significantly impairs physical health, while mental health is not significantly affected. Political identity, social justice cognition and job satisfaction all have a significant positive effect on physical and mental health. Among them, residents with party membership and high social justice cognition have better physical and mental health. The level of education is only positively correlated with physical health, reflecting the role of education in promoting healthy behavior. It is worth noting that residence, religious belief and BMI index did not show the expected impact on physical and mental indicators. Overall, model 1 confirms the important role of social structural factors and subjective cognition in health.

Model 2 adds physical exercise factors on the basis of model 1. The results show that physical exercise has a significant positive impact on the health of residents, with a coefficient of 0.052 and significant at the 1% level, indicating that physical exercise can effectively improve the health of residents. But its role in promoting mental health did not reach a significant level. Compared with model 1, the coefficients and significance levels of most control variables do not change much, indicating that the introduction of physical exercise factors has little effect on the control variables of the original model, and the model has certain stability.

Model 3 adds social network factors on the basis of model 2. The results show that social network has a significant positive impact on residents’ physical health, with a coefficient of 0.081 and significant at the 1% level, indicating that a good social network can promote physical health. Social network has a significant positive impact on residents ‘mental health, with a coefficient of 0.067 and significant at the 5% level, indicating that good social network can promote mental health. Compared with model 2, after the introduction of social network factors, the coefficients of some variables may change slightly, but the significance and influence direction of each control variable are basically consistent, showing the robustness of the model.

Model 4 adds digital health literacy factors on the basis of model 3. The results show that digital health literacy has a significant positive impact on residents’ mental health. The coefficient is 0.752 and significant at the 1% level, which proves that the research hypothesis H1b is valid. It shows that digital health literacy can help residents better acquire and understand mental health knowledge, make timely psychological adjustment and seek help, so as to significantly improve their psychological state; digital health literacy has a significant positive impact on residents’ physical health, with a coefficient of 0.146 and significant at the 5% level, which proves that the research hypothesis H1a is valid. Compared with model 3, after the introduction of digital health literacy factors, the variable coefficients in the previous models changed little, and the significance level basically maintained the original state, indicating that the model had good stability. The addition of digital health literacy factors did not significantly interfere with the relationship between the original variables. Through collinearity diagnosis, the VIF value is less than 5, and the tolerance is greater than 0.2 (VIF<5), indicating that there is no collinearity problem (Tolerability>0.2), and there is no high correlation between independent variables, which proves that the hypothesis test of multiple linear regression model is reliable.

### 4.3 Robustness test results

In order to further confirm the robustness of the results of the impact of digital health literacy on residents’ physical and mental health, this study uses three methods to test, and the results are shown in Table 4. First, the principal component analysis method was used to re-evaluate digital health literacy. After testing, *p* = 0.000, KMO = 0.719, indicating that it is suitable for principal component analysis. The results are shown in model 5. Secondly, when the dependent variable is an ordinal variable with a category number greater than or equal to 5, the results of the continuous data analysis method are basically close to the hierarchical data estimation method. Therefore, this study uses the OLS estimation method for regression (48), and the results are shown in model 6. Third, the independent variable digital health literacy and the dependent variable physical and mental health are ordered variables. Therefore, this study uses the ordered Logit model for regression, and the results are shown in model 7.

**Table 4:** Robustness test results.

Variable	Model 5		Model 6		Model 7	
			OLS regression		Ordered Logit regression	
	Physical health	Mental health	Physical health	Mental health	Physical health	Mental health
Digital health literacy (PCA)	0.088* (0.014)	0.026** (0.015)				
Digital health literacy			1.013* (0.162)	0.464** (0.170)	1.798* (0.304)	0.948** (0.304)
Control variable	Has been controlled	Has been controlled	Has been controlled	Has been controlled	Has been controlled	Has been controlled
*N*	1805	1805	1805	1805	1805	1805
Constant	3.658** (0.023)	4.027** (0.024)	3.271** (0.066)	4.205** (0.069)		
Adjusted R^2^	0.021	0.024	0.021	0.004		

*p < 0.05, **p < 0.01, standard error in parentheses.

In Model 5, the regression coefficient of the impact of digital health literacy (PCA) on physical health was 0.088, and it was significant at the 5% significance level (*p* < 0.05), indicating that digital health literacy had a significant positive impact on physical health. The regression coefficient of the impact of digital health literacy (PCA) on mental health was 0.026, and it was significant at the 1% significance level (*p* < 0.01), indicating that the improvement of digital health literacy also had a significant positive effect on mental health. After the re-evaluation of digital health literacy by principal component analysis, it still has a significant role in promoting the physical and mental health of residents, and has good robustness.

In Model 6, the regression coefficient of the impact of digital health literacy on physical health was 1.013, and it was significant at the 5% significance level (*p* < 0.05), indicating that it had a significant effect on physical health without changing the evaluation method of digital health literacy. The regression coefficient of the impact of digital health literacy on mental health is 0.464, and it is significant at the 1% significance level (*p* < 0.01), indicating that digital health literacy also has a significant positive impact on mental health. This is consistent with the conclusion of Model 5, which further verifies the robustness of the positive impact of digital health literacy on physical and mental health.

In Model 7, the regression coefficient of the impact of digital health literacy on physical health is 1.798, and it is significant at the 5% significance level (*p* < 0.05), indicating that digital health literacy has a significant positive impact on physical health. The regression coefficient of the impact of digital health literacy on mental health is 0.948, and it is significant at the 1% significance level (*p* < 0.01), indicating that digital health literacy also has a significant positive effect on mental health. It shows that under different regression models, the promotion effect of digital health literacy on physical and mental health is still robust and significant.

Whether it is using principal component analysis to re-evaluate Model 5 of digital health literacy, traditional OLS regression Model 6 and ordered Logit regression Model 7, digital health literacy has a significant positive impact on residents’ physical and mental health. This fully demonstrates that the research results have strong robustness and do not change directionally or fundamentally due to different model choices, which strongly supports the conclusion that digital health literacy has a positive effect on residents’ physical and mental health.

### 4.4 Mediating effect results

Through Bootstrap analysis, this study deeply discusses whether digital health literacy and residents’ physical and mental health are mediated by physical exercise and social network. The results of mediating effect analysis are shown in Table 5.

**Table 5:** Bootstrap analysis of mediating effect.

Item	Effect	Boot SE	BootLLCI	BootULCI
Digital health literacy ⇒ Participating in the physical exercise ⇒ Physical health	0.113	0.005	0.007	0.027
Digital health literacy ⇒ Social networking ⇒ Physical health	0.035	0.002	0.001	0.010
Digital health literacy ⇒ Participating in the physical exercise ⇒ Mental health	0.126	0.005	0.007	0.030
Digital health literacy ⇒ Social networking ⇒ Mental health	0.033	0.002	0.001	0.010
Digital health literacy ⇒ Participating in the physical exercise ⇒ Social networking ⇒ Physical health	0.016	0.001	0.001	0.004
Digital health literacy ⇒ Participating in the physical exercise ⇒ Social networking ⇒ Mental health	0.015	0.001	0.001	0.004

BootLLCI refers to the lower limit of 95% interval of Bootstrap sampling, and BootULCI refers to the upper limit of 95% interval of Bootstrap sampling.

Participation in physical exercise and social network play an important intermediary role in the impact of digital health literacy on residents’ physical and mental health. As shown in Table 5, the effect of digital health literacy on physical health by participating in physical exercise is 0.113, the Bootstrap standard error is 0.005, and the 95% confidence interval is [0.007, 0.027], excluding 0, indicating that participating in physical exercise plays a significant intermediary role in the impact of digital health literacy on physical health, which proves that the research hypothesis H2a is established. The effect value of digital health literacy on physical health through social network is 0.035, Bootstrap standard error is 0.002, 95% confidence interval is [0.001, 0.010], excluding 0, indicating that social network plays a significant mediating role in the impact of digital health literacy on physical health, which proves that the research hypothesis H3a is established. The effect value of digital health literacy on mental health by participating in physical exercise is 0.126, the Bootstrap standard error is 0.005, and the 95% confidence interval is [0.007, 0.030], excluding 0, indicating that participating in physical exercise plays a significant mediating role in the impact of digital health literacy on mental health, which proves that the research hypothesis H2b is established. The effect value of digital health literacy on mental health through social network is 0.033, Bootstrap standard error is 0.002, 95% confidence interval is [0.001, 0.010], excluding 0, indicating that social network plays a significant mediating role in the impact of digital health literacy on mental health, which proves that the research hypothesis H3b is valid.

The chain mediating effect path was analyzed. According to the chain mediating path of “digital health literacy ⇒ participating in the physical exercise ⇒ social networking ⇒ physical health,” the effect value was 0.016, the Bootstrap standard error was 0.001, and the 95% confidence interval was [0.001, 0.004], excluding 0, indicating that there was a chain mediating effect, which proved that the research hypothesis H4a was established. According to the chain mediating path of “digital health literacy ⇒ participating in the physical exercise ⇒ social networking ⇒ mental health,” the effect value is 0.015, the Bootstrap standard error is 0.001, the 95% confidence interval is [0.001, 0.004], excluding 0, indicating that there is a chain mediating effect, which proves that the research hypothesis H4b is established.

Overall, compared with other paths, the direct mediating effect of physical exercise (0.113 and 0.126) is the largest. This shows that physical exercise is a core and powerful intermediary mechanism for digital health literacy to improve physical and mental health. Although the independent mediating effect of social network (0.035 and 0.033) is smaller than that of physical exercise path, its confidence interval is also far from 0, indicating that its role is stable and statistically significant. The effect value of chain mediation (0.016 and 0.015) is the smallest among the three, but its existence reveals the complexity of the mechanism of action between variables. The results show that the impact of digital health literacy on residents’ physical and mental health is not single and direct, but through multiple parallel and interrelated intermediary paths. Physical exercise and social network play an independent and important intermediary role respectively, and there is also a linkage effect between the two (chain intermediary).

### 4.5 Heterogeneity analysis results

In order to further explore whether there are differences in the relationship between digital health literacy and residents’ physical and mental health between gender, place of residence and BMI index, this study constructs a grouping regression model, and divides the overall sample into groups according to male and female, urban and rural, BMI index wasting, normal and overweight. The results are shown in Tables 6, 7, 8.

**Table 6:** Gender heterogeneity results.

Variable	Physical health		Mental health	
	Female	Male	Female	Male
Digital health literacy	0.997** (0.220)	0.707** (0.250)	0.119 (0.238)	1.200** (0.246)
Control variable	Has been controlled	Has been controlled	Has been controlled	Has been controlled
*N*	989	816	989	816
Constant	3.446** (0.237)	4.021** (0.275)	4.495** (0.257)	4.732** (0.271)
Adjusted R^2^	0.037	0.031	0.019	0.027

*p < 0.05, **p < 0.01, standard error in parentheses.

**Table 7:** Urban-rural heterogeneity results.

Variable	Physical health		Mental health	
	Rural	Urban	Rural	Urban
Digital health literacy	1.193** (0.236)	0.423 (0.228)	0.511* (0.242)	0.816** (0.245)
Control variable	Has been controlled	Has been controlled	Has been controlled	Has been controlled
*N*	996	809	996	809
Constant	3.515** (0.250)	3.938** (0.257)	4.602** (0.257)	4.659** (0.277)
Adjusted R^2^	0.049	0.016	0.010	0.021

*p < 0.05, **p < 0.01, standard error in parentheses.

**Table 8:** BMI index heterogeneity results.

Variable	Physical health			Mental health		
	Emaciation	Normal value	Overweight	Emaciation	Normal value	Overweight
Digital health literacy	1.850** (0.608)	0.948** (0.217)	0.364 (0.274)	−0.380 (0.567)	0.574* (0.230)	−0.740* (0.295)
Control variable	Has been controlled	Has been controlled	Has been controlled	Has been controlled	Has been controlled	Has been controlled
*N*	164	1,002	639	164	1,002	639
Constant	3.535** (0.648)	3.781** (0.242)	3.661** (0.289)	5.132** (0.603)	4.760** (0.257)	4.255** (0.312)
Adjusted R^2^	0.131	0.033	0.013	0.011	0.017	0.016

*p < 0.05, **p < 0.01, standard error in parentheses.

As shown in Table 6, in terms of physical health, digital health literacy has a significant positive impact on women’s physical health, with a coefficient of 0.997 (*p* < 0.01). Digital health literacy has a significant positive impact on male health, with a coefficient of 0.707 (*p* < 0.01). However, women are more affected, which may be due to the fact that women pay more attention to health information and the improvement of digital health literacy brings more positive behavior changes. In terms of mental health, digital health literacy has a significant positive impact on male mental health, with a coefficient of 1.200 (*p* < 0.01), and has no significant impact on women (coefficient 0.119, and *p* > 0.05). Men may be more likely to use digital health literacy to obtain psychological adjustment methods, while women may be distracted by family responsibilities, which affects their mental health improvement.

As shown in Table 7, in terms of physical health, rural residents’ digital health literacy has a significant impact on physical health, with a coefficient of 1.193 (*p* < 0.01). The digital health literacy of urban residents has no significant effect on physical health (coefficient is 0.423, and *p* > 0.05). Rural medical resources are relatively scarce, and the improvement of digital health literacy can make up for the deficiency and improve health. Urban residents have multiple access to information, and the impact of digital health literacy on health is relatively weakened. In terms of mental health, rural and urban residents’ digital health literacy has a positive impact on mental health. The rural residents’ coefficient is 0.511 (*p* < 0.05), and the urban residents’ coefficient is 0.816 (*p* < 0.01). The psychological pressure of rural residents is great, and the help of digital health literacy is limited, but it has a certain mitigation effect. Urban residents use digital resources more efficiently and promote mental health more significantly.

As shown in Table 8, in terms of physical health, the digital health literacy of the thin group has a significant positive impact on physical health, with a coefficient of 1.850 (*p* < 0.01), the normal group is also significant (coefficient 0.948, and *p* < 0.01), and the overweight is not significant (coefficient 0.364, and *p* > 0.05). The thin and normal groups pay more attention to health information, and the improvement of literacy brings health improvement; overweight people or because of complex health problems, it is difficult to improve digital health literacy alone. In terms of mental health, the digital health literacy of the thin group had no significant effect on mental health (coefficient −0.380, and *p* > 0.05), the normal group had a positive effect (coefficient 0.574, and *p* < 0.05), and the overweight group had a negative effect (coefficient −0.740, and *p* < 0.05). The normal value group can effectively use digital health literacy to improve mental health; emaciation may have a psychological burden due to weight problems, weakening the impact; overweight people may have psychological pressure due to appearance and health problems, and digital health literacy is difficult to improve.

## 5 Discussion and conclusions

This study uses CGSS2021 data to empirically analyze the impact of digital health literacy on residents’ physical and mental health. The results show that:

1. Digital health literacy has a significant positive impact on residents’ physical and mental health, which is significant at the 5% level, which is consistent with the previous research results (49). After re-evaluating the dependent variables, replacing the model and other robustness tests, the research conclusions are still valid. Through Internet-based digital health literacy interventions, residents can quickly obtain relevant knowledge and methods to alleviate negative emotional symptoms such as anxiety and depression. These interventions often provide timely psychological support and coping strategies for residents, thus effectively weakening the occurrence and development of mental illness (50). In the long run, the improvement of digital health literacy helps to enhance residents’ critical thinking ability, so that they can view health information more objectively and rationally, and further transform the digital skills they have mastered into psychological resilience. This enhancement of psychological resilience can promote the long-term transformation of residents’ health behavior, so that they can maintain a more stable psychological state in the face of various pressures and challenges in life, so as to build a sustainable mental health maintenance ability (51). By improving residents’ digital health literacy, it is not only helpful for immediate psychological crisis intervention, but also for building sustainable physical and mental health maintenance capabilities. Based on this, residents should actively learn and improve their digital health literacy, make full use of the rich resources on the Internet, pay attention to the authoritative health information platform, and learn the correct physical and mental health knowledge and skills. At the same time, we should learn to distinguish the authenticity of information and avoid being misled by false information.
2. In terms of mediating effect. The study found that participation in physical exercise and social networks play a mediating role in the impact of digital health literacy on residents’ physical and mental health, and there is a chain mediating effect (95% confidence interval does not include 0). Specifically, digital health literacy has a positive impact on residents’ physical and mental health by promoting physical exercise and social networks. At the physical health level, digital health literacy indirectly improves physiological status by promoting physical exercise and expanding social networks (52); at the psychological level, through the Patient-to-Peer platform, residents can communicate and help others with similar health experiences on the platform, share their feelings and troubles, and obtain emotional comfort and understanding, so as to effectively relieve psychological pressure (53). In particular, the chain path reveals that digital health literacy first drives health behavior, then strengthens social connection, and finally enhances well-being and forms a virtuous circle (54). These findings indicate that improving digital health literacy can not only directly improve the physical and mental health of residents, but also indirectly have a positive impact by promoting health behavior and social interaction. Based on this, residents are encouraged to actively participate in physical exercise, use digital health literacy to obtain scientific exercise knowledge and methods, and formulate their own exercise plans and adhere to them. At the same time, it promotes the development of social interaction activities, and various organizations expand social support networks to provide strong support for residents’ mental health.
3. In terms of heterogeneity analysis. The study found that in rural areas and women with low or normal BMI, the improvement of digital health literacy has a significant effect on health promotion; in the male group of urban residents, the improvement of digital health literacy has a significant effect on mental health promotion. This differentiation mechanism is particularly prominent in the older adult group. The digital health literacy of the rural older adult is generally not high, and targeted intervention has become a breakthrough to improve the physical health of the group. In contrast, in urban environments, the positive impact of digital health literacy on mental health is more pronounced. This heterogeneity suggests that health promotion strategies need to develop differentiated programs based on geographical, gender and physiological characteristics, narrow the health information gap through rural digital skills training, and expand psychological support services based on urban digital platforms, so as to maximize the inclusive value of digital health literacy (55). Based on this, digital health literacy education can be incorporated into the national education system, and students’ digital health literacy can be cultivated from the school education stage to improve their ability to obtain, evaluate and apply health information. At the same time, a variety of digital health literacy training programs should be carried out for people of different ages and characteristics, especially to strengthen the training support for the older adult, low-educated people and other digital vulnerable groups. Through community education, older adult universities, online education and other ways, their digital skills and health management level can be improved.

This study preliminarily explores the impact of digital health literacy on the physical and mental health of residents, but there are still some deficiencies. First of all, based on cross-sectional data, this study cannot accurately determine the causal relationship, and equates instantaneous health fluctuations with relative stable literacy, which is easy to amplify or underestimate the real effect. In the future, longitudinal research design can be used to establish a 12–24-month multi-wave resident cohort, and latent variable growth curve and cross-lagged parallel strategy can be used to describe the two-way lag effect of literacy and health, so as to better dynamically explore the causal path between digital health literacy and physical and mental health. Secondly, the research samples may not fully represent the whole population, especially the groups with differences in the use of digital technology. Future research should expand the sample range and improve the diversity of samples. In addition, because of the increasing importance of digital health literacy, health information reliability and access are likely to become more relevant issues. We need to deepen the connotation of digital health literacy and further clarify the different dimensions of digital health literacy, such as information acquisition, information evaluation, information application, etc. And the specific performance and mechanism of these dimensions in different health situations. This helps to more accurately measure and analyze the relationship between digital health literacy and health outcomes.

## Data Availability

The datasets presented in this study can be found in online repositories. The names of the repository/repositories and accession number(s) can be found in the article/Supplementary material.

## Footnotes

^1^ http://www.cnsda.org/index.php?r=projects/view&id=65635422

## Appendix: Calculation of entropy weight TOPSIS method for digital health literacy

In order to better judge and measure the ranking of digital health literacy, this study uses the entropy weight TOPSIS method to determine the weight of digital health literacy indicators and calculate the score of digital health literacy. Entropy weight TOPSIS is a combination of entropy weight method (entropy method) and TOPSIS method, and its calculation is divided into two steps. The first step is to use the entropy weight method to calculate the weight value (see Table A1), and the data is weighted to obtain new data; the second step is to use the new data for TOPSIS method, and finally complete the analysis (see the uploaded EXCEL table ‘digital health literacy entropy weight TOPSIS calculation results’). In this study, the new data generated by the entropy weight TOPSIS method was used to measure the residents ‘digital health literacy, and the relevant regression analysis was carried out’.

**Table A1:** Weight of digital health literacy measurement index

**Variable**	**Survey questions used as measurement indicators**	**Information entropy e**	**Information utility value d**	**Weight coefficient w**
Digital health literacy	Do you often search for information about healthy lifestyles online?	0.9861	0.0139	20.1265%
	Do you often search for anxiety, stress-related or similar information online?	0.9844	0.0156	22.6100%
	Do you often search the Internet for information about vaccination?	0.9860	0.0140	20.3661%
	Does the information on the Internet have a positive impact on my health behavior?	0.9940	0.0060	8.7182%
	Does the information on the Internet help me understand the doctor's advice and treatment information?	0.9952	0.0048	6.9785%
	Does the Internet help to determine whether you need to go to the hospital?	0.9938	0.0062	8.9537%
	Does the Internet help to confirm whether the doctor has given appropriate advice?	0.9953	0.0047	6.8591%
	It is not easy to identify whether the health information on the Internet is reliable.	0.9963	0.0037	5.3878%
